# Dynamic Response of UHMW-PE Composite Armors under Ballistic Impact of Blunt Projectiles

**DOI:** 10.3390/ma15165594

**Published:** 2022-08-15

**Authors:** Li Ding, Xiaohui Gu, Peihui Shen, Xiangsheng Kong, Yi Zhou

**Affiliations:** 1 School of Mechanical Engineering, Nanjing University of Science and Technology, Nanjing 210094, China; 2 Nanjing Changjiang Electronics Group Co., Ltd., Nanjing 210037, China

**Keywords:** ordnance science and technology, UHMW-PE composite armors, dynamic response, ballistic impact, blunt projectile

## Abstract

To study the dynamic response of UHMW-PE composite armor under ballistic impact, two kinds of UHMW-PE composite armors are designed. Both of them are composed of UHMW-PE laminates and steel face sheets of Q235. The blunt projectile is made of 35CrMnSiA, with a cylinder shape. By numerical simulation, the dynamic response and deformation of composite armors are obtained under the penetration of the projectile. With the increase of impact velocity, the penetration depth increases nearly linearly, with a more severe tendency of swaging in the projectile. Then, experiments are carried out to validate the numerical simulation results. Based on a ballistic gun with a caliber of 14.5 mm, the projectiles are fired with a velocity from 680 m/s to 1300 m/s. The penetration into the composite armor can be divided into an initial shear plugging stage and the following bulging and delamination stage. Based on the theoretical analysis, the shear strength in the shear plugging stage can be estimated. Associated with typical experimental results, numerical simulation is suitable to predict the bulging characteristics of the composite armor. The failure mode of the composite armors under the impact of blunt projectiles is determined, and the failure mechanism is analyzed. The penetration results in the experiment agree well with the numerical simulation results, which validate the correctness of the numerical simulation models. The research results can be significant in the design of composite armor with UHMW-PE laminates.

## 1. Introduction

Polymer-based fiber-reinforced composites such as ultra-high molecular weight polyethylene (UHMW-PE) composites have gained more and more attention, and they are increasingly being employed in the defence industry to protect important structures from ballistic impact [1,2,3].

The ballistic performance of UHMW-PE has been studied both from experiments and numerical simulation. L. H. Nguyen et al. [4,5,6,7,8] proposed the numerical methodology for hydrocode analysis of UHMW-PE composite under ballistic impact and carried out experimental research to validate the results. Deflection and bulging, or a two-stage penetration process composed of shear plugging and the formation of a transition plane and bulging were the predominant failure modes of PE with different thicknesses under ballistic impact. Through fractographic observations on laminates, the determined sequence of failure modes is delamination, ply splitting and fibre kinking [9]. Based on the principle of conservation of energy, the relationship between deformation and energy dissipation of PE [10] and an analytical model to predict the ballistic limit of the PE laminate [2] were studied.

Sandwich structures consisting of thin face sheets and low-density non-metal cores have been widely studied [11,12] and can provide reference and methodology in the application of UHMW-PE. UHMW-PE has been used as part of other composite armors such as 30CrMnMo-UHMWPE Composite Armor [13], metal/UHMWPE/SiC multi-layered composite [14], Ceramic/UHMWPE Armors [15], etc., [16].

Following on from these findings, there is still limited report and understanding of the dynamic response of composite armors of UHMW-PE laminates and steel sheets. In this paper, two kinds of UHMW-PE composite armors are designed; both of them are composed of UHMW-PE laminates and steel face sheets of Q235. By numerical simulation and experimentation, the dynamic response of UHMW-PE composites armor under typical ballistic impact is investigated.

## 2. Design of the Armor and Projectile

**Figure 1:**
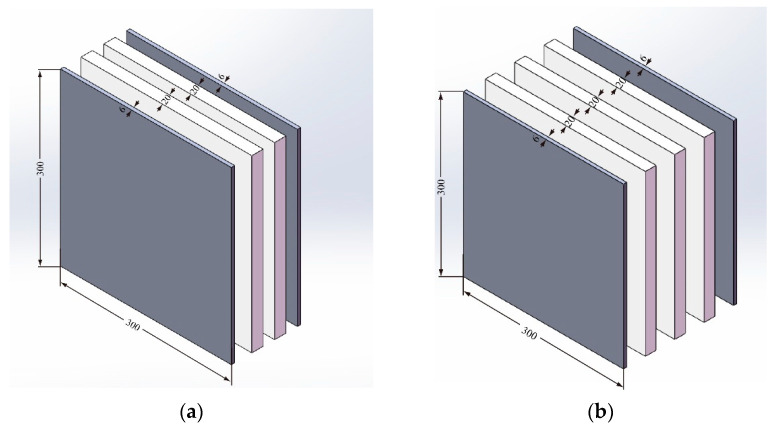
Structures of UHMW-PE composite armors. (**a**) Armor with two layers of PE. (**b**) Armor with three layers of PE.

There are two types of UHMW-PE composite armors being developed, both of which are made up of inner UHMW-PE laminates and steel face sheets. For reasonably acceptable strength, low price and wide availability, Q235 steel is selected as the face and back sheets. Typical UHMW-PE laminate with a material grade of FDB4-HW-S1 is also selected. The thickness of the UHMW-PE laminate remains constant at 20 mm; however, the thickness of the two front and back face sheets is 6 mm, as shown in Figure 1. The structure of armor with two layers of UHMW-PE is shown in Figure 1a, and the structure of armor with three layers of UHMW-PE is shown in Figure 1b. Each layer of armor has the same in-plane dimension of 300 mm × 300 mm. The two varieties of UHMW-PE composite armors have total thicknesses of 52 mm and 72 mm, respectively. Due to the existence of a binder layer between the PE and steel sheet, the total thickness of each type of armor may increase by 1 mm. Table 1 show the material properties of Q235 steel.

**Figure 2:**
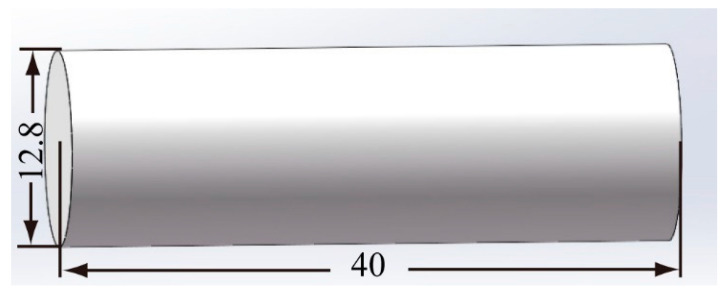
Structure of the blunt projectile.

The structure of a blunt projectile with a diameter of 12.8 mm, a height of 40 mm and a mass of 40 g is shown in Figure 2. The projectile is made of 35CrMnSiA.

## 3. Numerical Simulation and Analysis

### 3.1. Setup of Numerical Model

To understand and predict the dynamic response of UHMW-PE composite armor under ballistic impact, three-dimensional numerical models are developedd using AUTODYN non-linear software. The version of AUTODYN is v11.0 in software of ANSYS 11.0, located in Nanjing, China.

**Figure 3:**
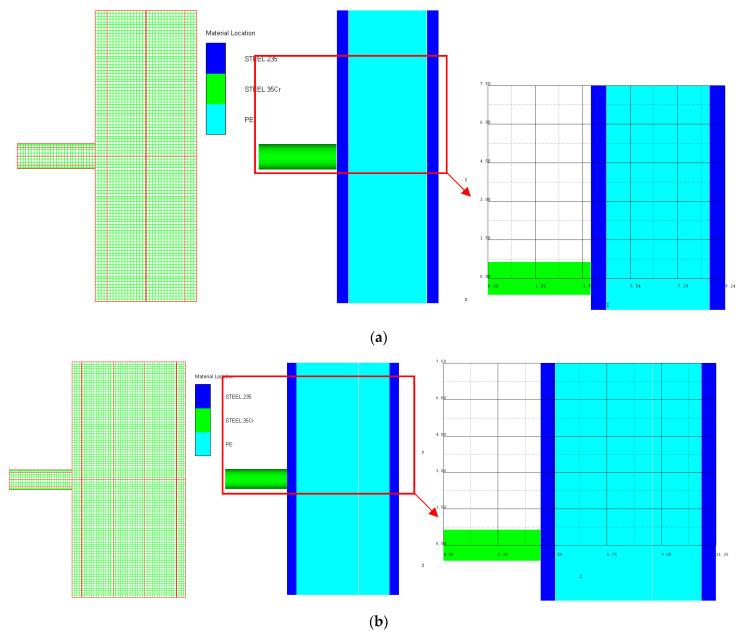
Grid and numerical models of armor impacted by blunt projectile. (**a**) Armor with two layers of UHMW-PE. (**b**) Armor with three layers of UHMW-PE.

As shown in Figure 3, all of the components in the numerical simulation are modeled with the 3D Lagrange algorithm in AUTODYN. Combing computational efficiency and accuracy, the half 3D model is carried out, with a mesh size of 1.2–1.5 mm per grid. With the grid size, the numerical models could yield acceptable accuracy with reasonable simulation time. The mesh is shown in the grid model on the left, and the numerical model is shown on the right. Fixed boundaries are deployed on the edge of the target. Different initial velocities are applied to the flat nose projectile to simulate penetration behavior with different velocities.

The material models of the projectile, face sheet and UHMW-PE laminate are listed in Table 2. In the numerical models, the shock equation of state, also called Grüneisen, is employed in conjunction with the Johnson–Cook constitutive model to simulate the dynamic response of the projectile and the face sheet. The Grüneisen EOS [17] can be used to describe how the materials interact with the shock wave and is based on Hugoniot’s relation between the *v*_s_ and the *v*_p_, as *v*_s_ = *c*_0_ + *sv*_p_, where *v*_s_ is the shock wave velocity, *v*_p_ is the material particle velocity, *c*_0_ is the wave speed and *s* is a material-related coefficient. The expression of the equation of the state of Grüneisen for the compressed state is:

(1) $$P=\frac{\rho_{0}C^{2}\mu \left[1+\left(1-\frac{\gamma_{0}}{2}\right)\mu -\frac{a}{2}\mu^{2}\right]}{\left[1-\left(S_{1}-1\right)\mu -S_{2}\frac{\mu^{2}}{\mu +1}-S_{3}\frac{\mu^{3}}{{\left(\mu +1\right)}^{2}}\right]}+\left(\gamma_{0}+a\mu \right)E.$$

In the expanded state,

(2) $$P=\rho_{0}C^{2}\mu +\left(\gamma_{0}+a\mu \right)E$$

where *C* is the intercept of velocity curve between shock wave and particle, *S*_1_, *S*_2_, and *S*_3_ represent the slope of the *v*_s_ − *v*_p_ curve, *γ*_0_ is the coefficient of Grüneisen and *a* is the one-order correction of *γ*_0_. *μ* = *ρ*/*ρ*_0_ − 1 is a non-dimensional coefficient based on initial and instantaneous material densities. The parameters of the Grüneisen equation of state are listed in Table 3.

The Johnson–Cook model [18,19] is a widely used constitutive model which incorporates the effect of strain rate-dependent work hardening and thermal softening. The Johnson–Cook constitutive relation is provided by:

(3) $$\sigma =\left(A+B\varepsilon^{n}\right)\left(1+C\ln\frac{\dot{\varepsilon }}{{\dot{\varepsilon }}_{0}}\right)\left(1-T^{*m}\right)$$

where *ε* is the plastic strain, and the temperature factor is expressed as:

(4) $$T^{*}=\frac{T-T_{r}}{T_{m}-T_{r}}$$

where *T_r_* is the room temperature, and *T_m_* is the melt temperature of the material. *A*, *B*, *n*, *C* and *m* are material-related parameters. The material parameters of 35CrMnSiA Q235 steel are presented in Table 4.

The orthotropic material model proposed by Long H. Nguyen et al. [5] was used for modeling the dynamic behavior of the UHMWPE layer subjected to ballistic impact. The correctness and accuracy were validated by Pengcheng Hu et al. [15]. The material model consists of a non-linear equation of state of orthotropic, a strength model and a failure model. The constitutive response of the material in the elastic regime is described as the orthotropic EOS composed of volumetric and deviatoric components. The pressure is defined by:

(5) $$\begin{array}{l} P=P\left(\varepsilon_{vol},e\right)-\frac{1}{3}\left(C_{11}+C_{21}+C_{31}\right)\varepsilon_{11}^{d}-\frac{1}{3}\left(C_{12}+C_{22}+C_{32}\right)\varepsilon_{22}^{d}\\ -\frac{1}{3}\left(C_{13}+C_{23}+C_{33}\right)\varepsilon_{33}^{d} \end{array}$$

where *C_ij_* are the coefficients of the stiffness matrix. $\varepsilon_{ij}^{d}$ refer to the deviatoric strains in the principal directions. The volumetric component $P\left(\varepsilon_{vol},e\right)$ is defined by the Mie-Grüneisen EOS:

(6) $$P\left(\varepsilon_{vol},e\right)=P_{r}\left(v\right)+\frac{\Gamma \left(v\right)}{v}\left[e-e_{r}\left(v\right)\right]$$

where, *v*, *e* and Γ(*v*) represent the volume, internal energy and the Grüneisen coefficient, respectively. *P_r_*(*v*) is the reference pressure, and *e_r_*(*v*) is the reference internal energy. The quadratic yield surface was adopted as the material strength model to describe the non-linear, irreversible hardening behavior of the composite laminate:

(7) $$\begin{array}{l} f\left(\sigma_{ij}\right)=a_{11}\sigma_{11}^{2}+a_{22}\sigma_{22}^{2}+a_{33}\sigma_{33}^{2}+2a_{12}\sigma_{11}\sigma_{22}+2a_{23}\sigma_{22}\sigma_{33}\\ +2a_{13}\sigma_{11}\sigma_{33}+2a_{44}\sigma_{23}^{2}+2a_{55}\sigma_{31}^{2}+2a_{66}\sigma_{12}^{2}=k \end{array}$$

where *a_ij_* are the plasticity coefficients, and *σ_ij_* represents the stresses in the principal directions of the material. Furthermore, the state variable, *k*, is used to define the border of the yield surface. It is described with a master effective stress-effective plastic strain curve defined by 10 piecewise points to consider the effect of strain hardening.

In the numerical models, the failure model of the orthotropic material is based on a combined stress criterion presented as follows:

(8) $${\left(\frac{\sigma_{ii}}{S_{ii}\left(1-D_{ii}\right)}\right)}^{2}+{\left(\frac{\sigma_{ij}}{S_{ij}\left(1-D_{ij}\right)}\right)}^{2}+{\left(\frac{\sigma_{ki}}{S_{ki}\left(1-D_{ki}\right)}\right)}^{2}\geq 1\;\mathrm{for}\;i,j,k=1,2,3$$

where *S_ii_* is the failure strength in the respective directions of the material, and *D_ii_* is the damage parameter following a linear relationship with stress and strain as below:

(9) $$D_{ii}=\frac{L\sigma_{ii,f}\varepsilon_{cr}}{2G_{ii,f}}$$

where *L* is the characteristic cell length, *ε_cr_* refers to the crack strain, and *G_ii_*_,*f*_ presents the fracture energy in the direction of damage.

The corresponding parameters of the material model for the orthotropic equation of state are provided in Table 5, and material constants for orthotropic yield strength are listed in Table 6.

### 3.2. Numerical Results and Analysis

Table 7 present the numerical simulation results of the blunt projectile penetrating the composite armor with two layers of PE. *v* is the impact velocity of the blunt projectile, and *p* is the depth of penetration. With the increase of impact velocity, the penetration depth increases gradually, and the projectile will have a more severe tendency to swage after penetration. After impact, due to the reflection of stress waves in the penetration process, the steel sheet and PE laminates may separate away from each other. The penetration depth *p* is measured from the head of the projectile to the baseline of the front sheet at the end of the simulation.

**Figure 4:**
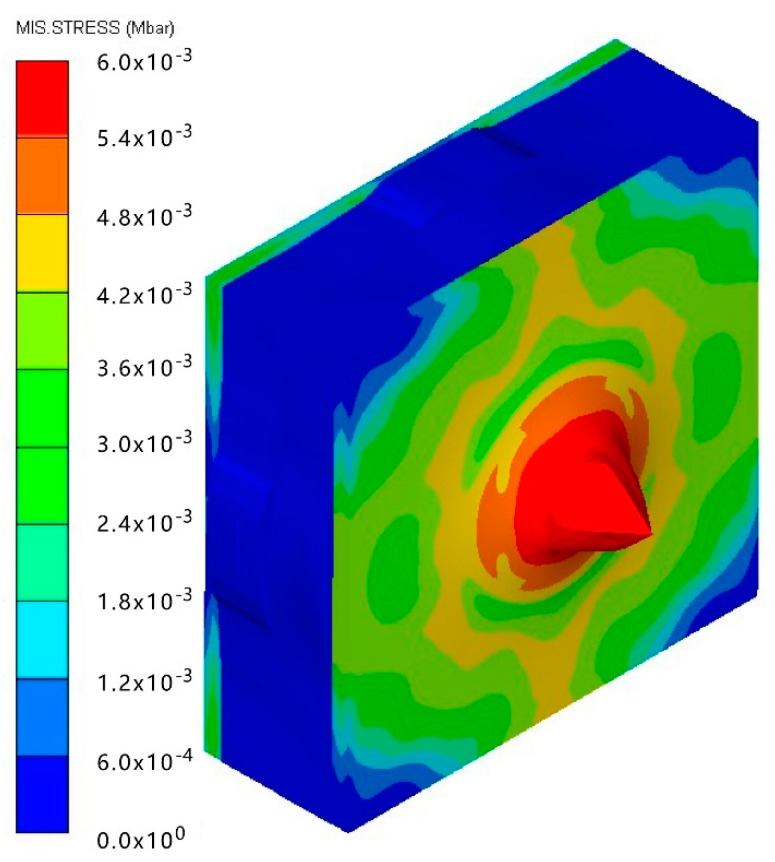
Von-Mises stress contour of armor at the impact velocity of 1300 m/s.

As shown in Table 7, when the impact velocity reaches 1300 m/s, the projectile will pass through the armor. As shown in Figure 4, the Von-Mises stress contour of the back sheet can be solid evidence to predict the failure of the steel sheet and perforation of the armor.

Table 8 present the numerical simulation results of the blunt projectile penetrating the composite armor with three layers of PE. With the increase of impact velocity, the penetration depth increases gradually. The projectile will have a more severe tendency of swaging. When the impact velocity exceeds 1000 m/s, the back sheet deforms severely and separates away from the PE laminates, mainly due to the reflection of stress wave in the penetration process within the interaction with different layers.

**Figure 5:**
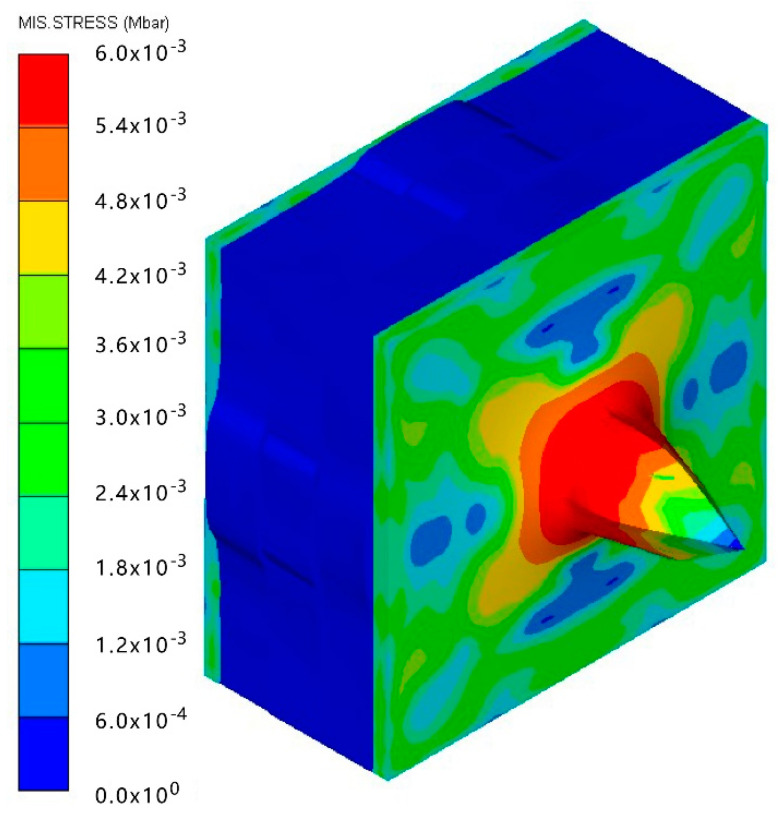
Von-Mises stress Contour of armor at the impact velocity of 1400 m/s.

As shown in Table 8, when the impact velocity reaches 1400 m/s, the projectile will pass through the armor. As presented in Figure 5, the Von-Mises stress contour of the back sheet can be solid evidence to predict the failure of the steel sheet and perforation of the armor with three layers of PE laminates.

It can be concluded from the numerical simulation: (1) the established numerical simulation models for the composite armors are able to predict the penetration and deformation of the target. (2) With the increase of impact velocity, the penetration depth increases gradually both for the armors with two and three layers of PE. (3) By numerical simulation, at the velocity of 1300 m/s, the blunt projectile could penetrate through the composite armor with two layers of PE. While at the velocity of 1400 m/s, the blunt projectile could penetrate through the composite armor with three layers of PE.

## 4. Experimental Details and Results

### 4.1. Design of the Experiment

**Figure 6:**
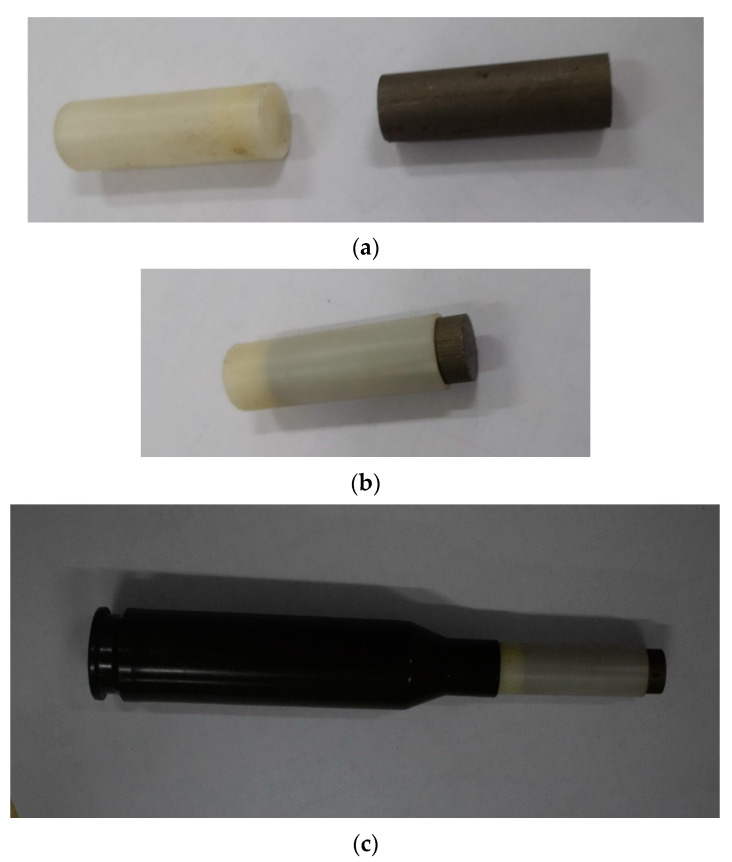
Photograph of the projectile in the test. (**a**) The projectile and sabot. (**b**) Assembly of the projectile in the sabot. (**c**) Assembly of the projectile in the shell case.

The state of the projectile in the test is presented in Figure 6. The sabot is designed to meet the launch requirements with nylon material. The state of the sabot and blunt projectile is presented in Figure 6a. As shown in Figure 6b,c, the projectile was firstly assembled in the sabot and then assembled in the shell case together with the sabot. The blunt projectile was fired from a 14.5 mm caliber ballistic gun. When the structure and mass of the projectile stay constant, the muzzle velocity of the projectile usually has a linear relationship with the mass of the propellant within a certain range. Thus, by adjusting the mass of propellant in the shell case, the required velocities of the projectile can be obtained.

**Figure 7:**
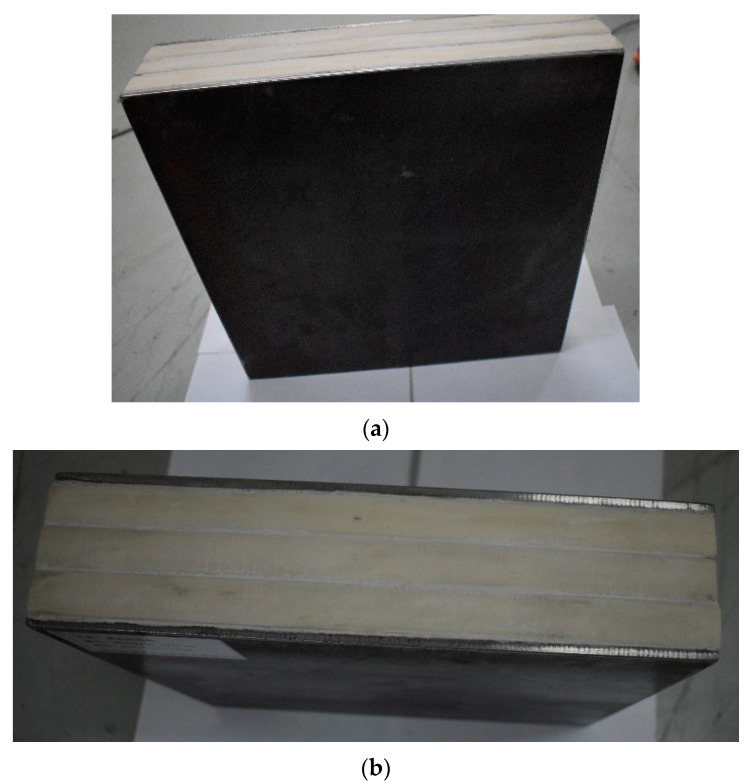
Photograph of the armors in the test. (**a**) Front view. (**b**) Side view.

**Figure 8:**
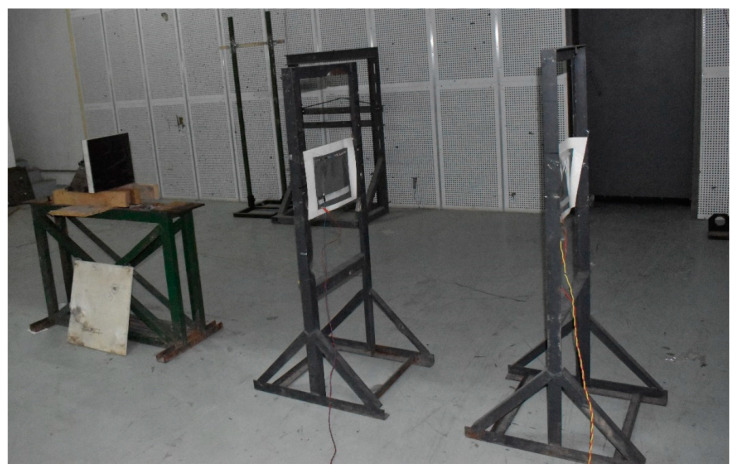
Layout of the ballistic impact experiment.

Figure 7 show the states of the armors used in the test. The armors were clamped to the rear base on the steel shelf. Two tinfoil targets were placed in front of the armor to measure the initial velocity of the projectile. The layout of the ballistic impact experiment is presented in Figure 8.

### 4.2. Experimental Results

Table 9 show the ballistic impact results of armors with two layers of PE, with typical ballistic velocity ranges from 700 m/s to 1200 m/s. Specifically, with the velocity from 759 m/s to 1174 m/s, the blunt projectile could not perforate the armor with two layers of PE. Only deformation and bulging with different degrees occurred.

**Figure 9:**
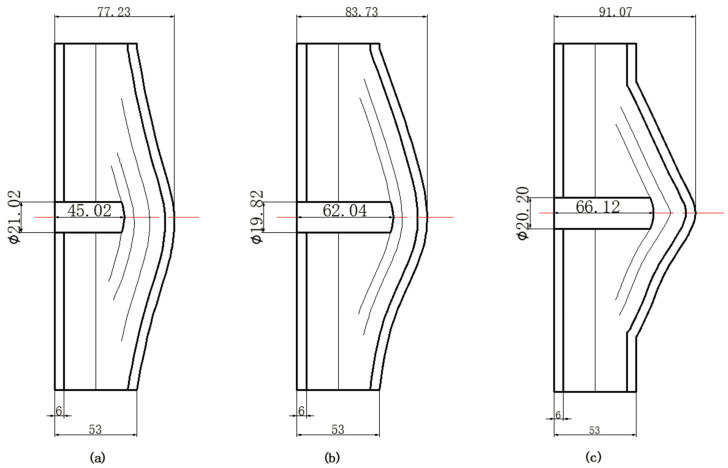
Deformation and perforation profiles of armor with two layers of PE (unit: mm). (**a**) *v* = 759 m/s. (**b**) *v* = 1139 m/s. (**c**) *v* = 1174 m/s.

The perforation dimensions are listed in Table 10. Within the velocity range from 760 m/s to 1174 m/s, the aperture diameter stayed around 20 mm. At the velocity of 1174 m/s, the depth of penetration ranged from 64 to 66 mm. The value of 66.12 mm was adopted as the penetration result of impact velocity of 1174 m/s, which can be drawn in a 2D drawing. It was concluded that with the increase of penetration depth, the bulging deformation grows. The deformation and perforation profiles of armor with two layers of PE are presented in Figure 9.

It can be concluded from Table 9 and Figure 9 that penetration into the composite armor can be divided into a two-stage process [4]: (1) an initial shear plugging stage, where there is little deflection of the target. (2) This is followed by the bulging or breakout of a sub-laminate. With large deformation and bulging, the delamination may extend to the edge of the PE laminate, which results in some sub-laminates separating and breaking the PE laminate into multiple pieces. With the increase of bulging and deformation, the depth of penetration may exceed the initial total thickness of the composite armor of 53 mm.

**Figure 10:**
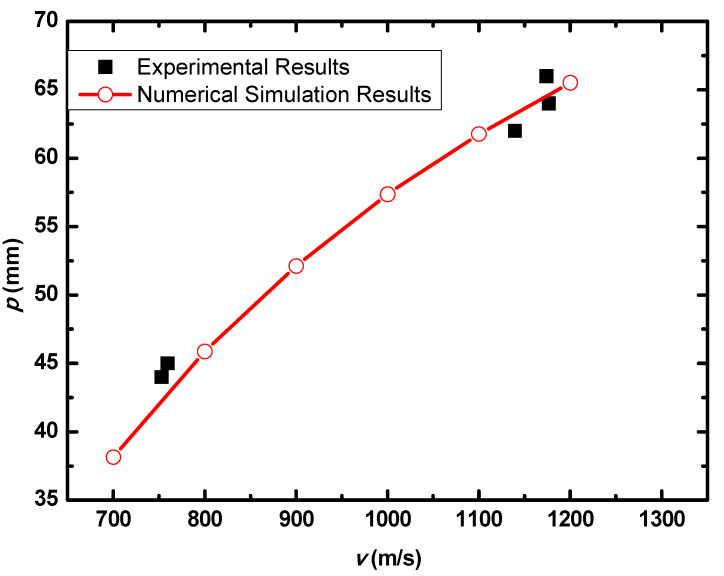
*p–v* curve of the blunt projectile penetration into the armor with two layers of PE.

With the increase of impact velocity, the penetration depth increases gradually and nearly linearly, which is presented in Figure 10. The numerical simulation results agree well with the experimental results.

Table 11 show the ballistics impact results of armors with three layers of PE, with the impact velocity ranging from 683 m/s to 1304 m/s. The velocity range is similar to the values in Table 9. As the projectile in tests No.2 and No.4 turned over with large angles of attack, the penetration results are not considered. With the velocity range from 683 m/s to 1304 m/s, the blunt projectile could not perforate the armor with three layers of PE. Only deformation and bulging with different degrees occurred. The perforation dimensions are listed in Table 12; the aperture diameter remained around 21 mm.

**Figure 11:**
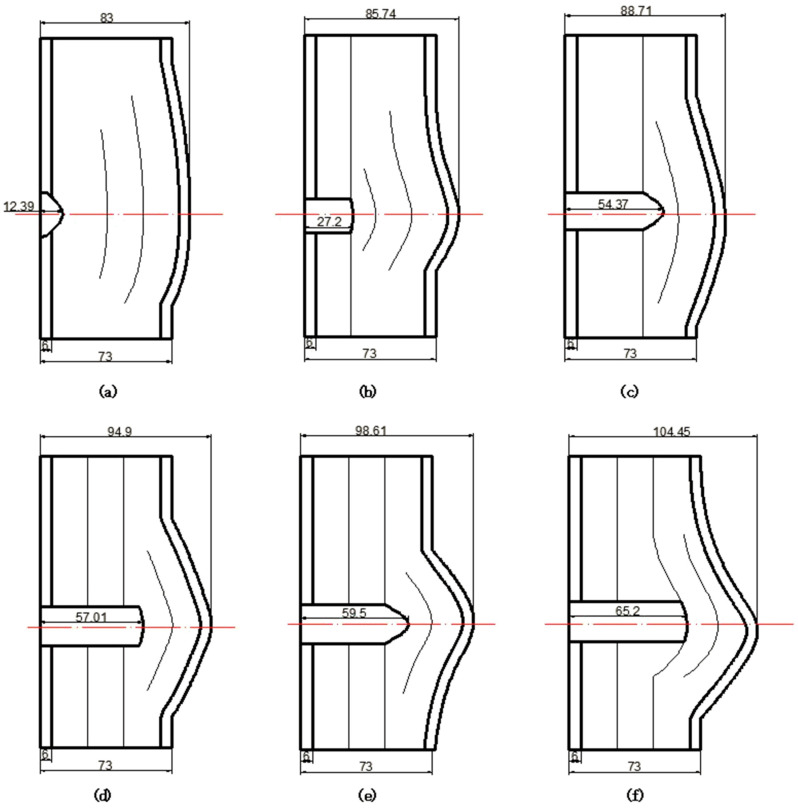
Deformation and perforation profiles of armor with three layers of PE (unit: mm). (**a**) *v* = 683 m/s. (**b**) *v* = 778 m/s. (**c**) *v* = 1175 m/s. (**d**) *v* = 1190 m/s. (**e**) *v* = 1127 m/s. (**f**) *v* = 1304 m/s.

**Figure 12:**
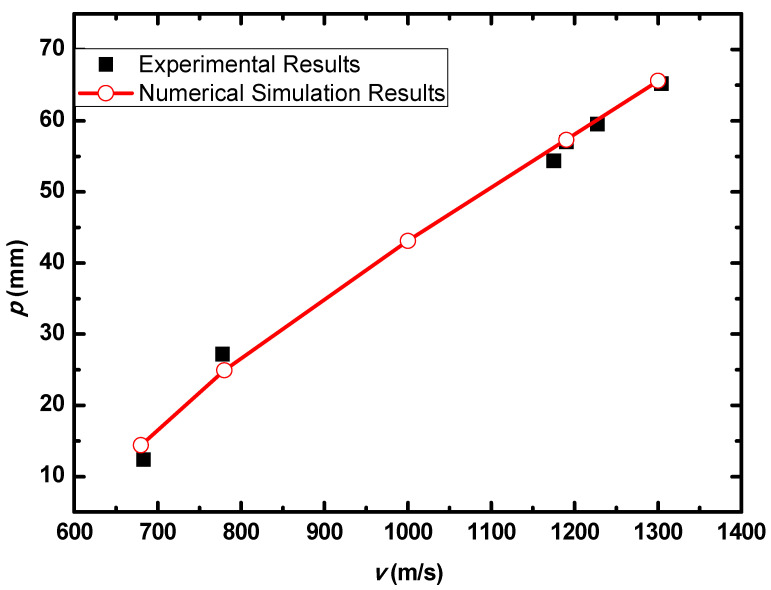
*p–v* curve of the blunt projectile penetration into the armor with three layers of PE.

Figure 11 show the deformation and perforation profiles of armor with three layers of PE. Associated with Table 9 and Figure 11, it can be concluded that the two-stage process in penetration still applies here. The transition between the two penetration stages is a complex physical phenomenon, and it has been proposed that transition is mainly due to delamination induced by shear-dominated stresses in bending [9]. According to the projectiles’ penetration states in the test, the penetration results are considered in the analysis, except for tests No.2 and No.4. The *p*–*v* curve of the blunt projectile penetration into the armor with three layers of PE is presented in Figure 12. With the increase of impact velocity, the penetration depth increases almost linearly, and the numerical simulation results agree quite well with the experimental results from the velocity of 683 m/s to 1304 m/s.

### 4.3. Discussion and Analysis

Penetration into the composite armor can be divided into a two-stage process: (a) an initial shear plugging stage; (b) the following bulging and delamination stage. Before penetrating through the composite armor, the kinetic energy of the projectile is assumed to be equal to the energy absorbed during the two-stage process, so

(10) $$E_{\mathrm{total}}=E_{S}+E_{B}=\frac{1}{2}m_{p}v_{i}{}^{2}$$

where $E_{\mathrm{total}}$ is the total kinetic energy of the projectile, *m*_p_ is the mass of the blunt projectile, *v*_i_ is the impact velocity of the projectile, *E_S_* is the energy absorbed in shear plugging and *E_B_* is the energy absorbed in the bulging stage.

**Figure 13:**
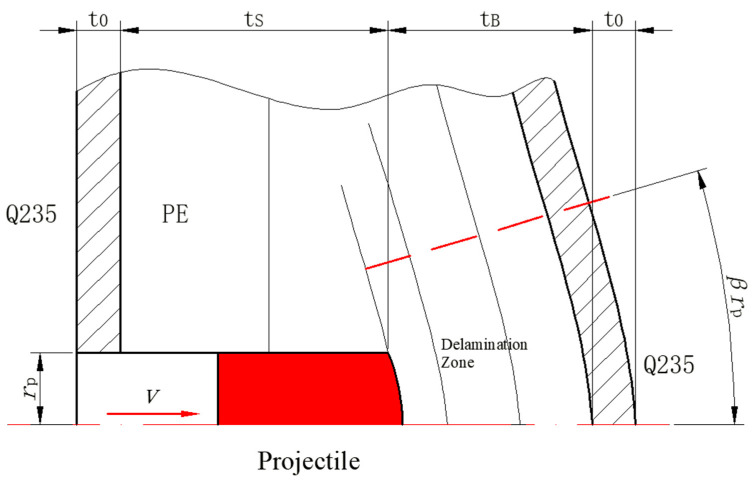
Schematic diagram of the two-stage penetration composed of shearing and bulging stages.

Figure 13 show the schematic diagram of the two-stage penetration composed of shearing and bulging stages. In the first penetration stage, the energy absorbed in shear plugging is equal to the work required to produce a shear plug composed of Q235 steel and partial PE laminate around the circumference of the blunt projectile, where the shear area is the product of the perimeter and the thickness of the material in the shear plugging process, which can be expressed by

(11) $$E_{S}=\int_{0}^{t_{s}}\tau_{\max}\left(2\pi r_{p}\right)\mathrm{tdt}=\tau_{Q235}\pi r_{p}t_{0}^{2}+\tau_{\max}\pi r_{p}t_{S}^{2}$$

where $\tau_{Q235}$ is the shear strength of steel Q235, $\tau_{\max}$ is the effective through-thickness shear strength of the laminate, *r*_p_ is the radius of the hole and β is a non-dimensional multiplier larger than the projectile radius, t_0_ is the thickness of the front sheet and t*_S_* is the PE thickness penetrated through shear plugging. By assuming penetration by transverse shearing only, the thickness of the plug is equal to the measured depth of penetration [4], then

(12) $$\frac{1}{2}m_{p}v_{i}{}^{2}=\tau_{\max}\pi r_{p}p^{2}+\tau_{Q235}\pi r_{p}{\left(p{-t}_{0}\right)}^{2}.$$

Equation (12) can be used to obtain the effective through-thickness shear strength using the test results, and the calculated data of shear strength $\tau_{\max}$ is presented in Table 13. For the composite armor with two layers of PE, when the impact velocity *v* exceeds 1139 m/s, the shear strength $\tau_{\max}$ will stabilize at about 13 GPa. In contrast, for the composite armor with three layers of PE, when the impact velocity *v* exceeds 1175 m/s, the shear strength $\tau_{\max}$ will reach a stable value of around 15 GPa. The calculated effective shear strengths are much higher than the laminate under ballistic impact without a steel sheet in the front and back, which are calculated to be from 388 MPa to 657 MPa [4]. This may be due to the constraining effect of the Q235 steel sheet, which enhances the armors’ resilience under ballistic impact.

In the second penetration stage, based on the conservation of momentum, the momentum of the projectile before bulging equals the total momentum of the projectile and the deformed zone of the armor. As shown in Figure 13, the momentum can be expressed as

(13) $$m_{p}v_{B}=\left(m_{p}+m_{B}+m_{0}\right)v_{}$$

(14) $$v_{B}=v\left(1+\frac{m_{B}}{m_{p}}+\frac{m_{0}}{m_{p}}\right)=v\left(1+\beta^{2}\frac{\pi r_{p}t_{B}\rho_{\mathrm{PE}}}{m_{p}}+\beta^{2}\frac{\pi r_{p}t_{0}\rho_{Q235}}{m_{p}}\right)$$

where *v_B_* is the projectile velocity before bulging, *v* is the velocity of the combined projectile and bulging mass of PE and steel sheet and *m_B_* and *m*_0_ are the mass of the target involved in the bulging stage of PE and back sheet. The energy absorbed in the bulging stage can be provided by

(15) $$E_{B}=\frac{1}{2}m_{p}v_{B}{}^{2}.$$

**Figure 14:**
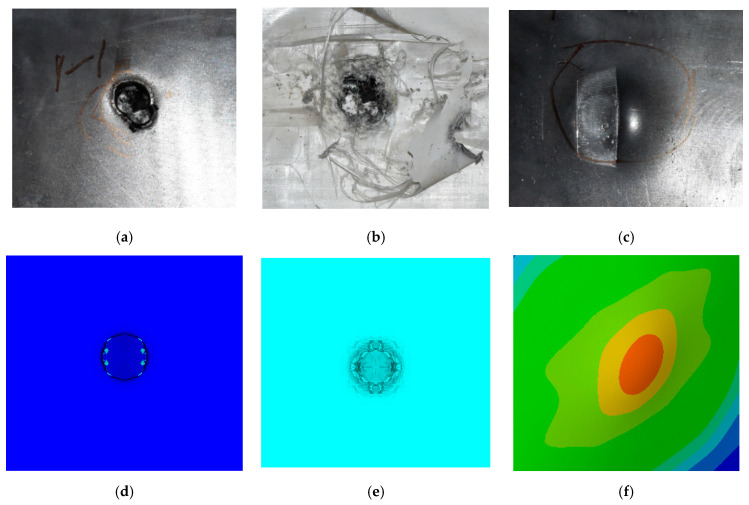
Comparison between the results of the experiment and the numerical simulation at the impact velocity of 759 m/s for composite armor with two layers of PE. (**a**) Face sheet. (**b**) Sub-laminate. (**c**) Deformation on the back sheet. (**d**) Face sheet. (**e**) Sub-laminate. (**f**) Deformation on the back sheet.

Due to the energy transferred in the bulging stage, the PE laminate and the back sheet deform severely and are able to resist the penetration of the blunt projectile at a rather high velocity. As the complexity phenomenon in bulging, numerical simulation associated with typical experimental results is suitable to predict the bulging characteristics of the composite armor. As shown in Figure 14, the perforation and deformation properties of (d), (e) and (f) in the numerical simulation match well with the experimental results of (a), (b) and (c).

By comparing the penetration results of two types of armors, it can be concluded that: (1) the sabot is designed to meet the launch requirements, which could be used in the ballistic gun to launch the blunt projectile with a velocity range of 680 m/s to 1300 m/s. (2) The two kinds of designed armors could be used to resist the impact of a blunt projectile, even at a velocity of 1170 m/s. By comparison, the armor with two layers of PE can be enough to resist the impact of a blunt project under the velocity of 1174 m/s. In contrast, the armor with three layers of PE can be enough to resist the impact of a blunt project under the velocity of 1304 m/s. (3) With the increase of impact velocity, the penetration depth increases gradually both for the armor of two layers and three layers of PE. (4) The penetration into the composite armor can be divided into an initial shear plugging stage and the following bulging and delamination stage. (5) Based on the experimental results, it may improve delamination-induced shear stress conditions to render a safer transition without deep penetration by increasing the shear strength and bond strength of the PE laminates. The failure mechanism of the composite armor is analyzed by theoretical models; based on the theoretical analysis, the through-thickness shear strength can be estimated, and numerical simulation associated with typical experimental results is suitable to predict the bulging characteristics of the composite armor.

## 5. Conclusions

Two types of multi-layered composite armors made up of inner UHMW-PE laminates and steel face sheets were proposed for the protection of important structures in the defence industry. A study of the dynamic response of UHMW-PE composite armor under typical ballistic impact was carried out. The conclusion can be obtained below:

(1) Based on a 14.5 mm caliber ballistic gun, two types of UHMW-PE composite armors were designed; both of them are composed of UHMW-PE laminates and steel face sheets of Q235. The nylon sabot was designed to meet the launch requirements, which could be used to launch the blunt projectile with a velocity range of 680 m/s to 1300 m/s.
(2) The established numerical simulation models for the composite armors were able to predict the penetration and deformation of the target. Using the orthotropic equation of state and Orthotropic yield strength model, a numerical model can be set up to simulate the dynamic response of UHMW-PE laminate under the ballistic impact of a blunt projectile. According to numerical simulation results, the blunt projectile was able penetrate through the composite armor with two layers of PE at a velocity of 1300 m/s, and it could penetrate through the composite armor with three layers of PE at a velocity of 1400 m/s.
(3) The two kinds of designed armors could be used to resist the impact of a blunt projectile even at a velocity of 1170 m/s. By comparison, the armor with two layers of PE can be enough to resist the impact of a blunt project under the velocity of 1174 m/s. At the same time, the armor with three layers of PE can be enough to resist the impact of a blunt project under the velocity of 1304 m/s.
(4) The failure mode of the composite armor can be determined, and the penetration into the composite armor can be divided into an initial shear plugging stage and the following bulging and delamination stage. With the increase of impact velocity, the penetration depth increases gradually both for the armor of two layers and three layers of PE. The projectile will have a more severe tendency of swaging.
(5) The failure mechanism of the composite armor was analyzed by theoretical models of a two-stage process. Based on the theoretical analysis, the through-thickness shear strength was estimated, and numerical simulation associated with typical experimental results were suitable to predict the bulging characteristics of the composite armor.

The numerical and experimental results provide necessary data support for the analysis of composite structure dynamic response under fragment impact and verify the correctness of the numerical simulation method. The research results are significant in the design of composite armor with UHMW-PE laminates. By combining steel face sheets and UHMW-PE laminates, it is possible to obtain composite armor that is good enough to resist the penetration of blunt projectiles.

## Figures and Tables

**Table 1:** Material properties of Q235 steel.

Steel	Yield Strength (MPa)	Tensile Strength (MPa)	Elongation after Break (%)	Poisson’s Ratio (%)	Impact Energy Aku (J)
Q235	305	426	30	0.33	≥27

**Table 2:** Material models used in numerical simulation.

Components	Material	*ρ* (g/cm^3^)	Equation of State	Constitutive Model
Projectile	35CrMnSiA	7.83	Shock	Johnson–Cook
Face sheet	Q235	7.896	Shock	Johnson–Cook
PE laminates	UHMW-PE	0.98	Ortho	Orthotropic Yield

**Table 3:** Parameters of Grüneisen equation of state 35CrMnSiA and Q235.

Material	Grüneisen Coefficient	*C* (m/s)	*S*_1_	*S*_2_	*a*
35CrMnSiA	2.02	3490	1.489	0	0.47
Q235	2.17	4569	1.490	0	0.46

**Table 4:** Material constants for 35CrMnSiA and Q235.

Steel	*ρ* (g/cm^3^)	*A* (MPa)	*B* (MPa)	*n*	*C*	*m*	${\dot{\varepsilon }}_{0}\;(s{-}^{1})$	*T_r_* (K)	*T_m_* (K)
35CrMnSiA	7.83	792	510	0.26	0.014	1.03	1	293	1793
Q235	7.896	350	275	0.36	0.022	1.00	1	293	1793

**Table 5:** Material constants for orthotropic equation of state.

Parameters	Value	Units	Parameters	Value	Units
Reference density	0.98	g/cm^3^	Shear modulus 12	2.0 × 10^6^	kPa
Young’s modulus 11	3.62 × 10^6^	kPa	Shear modulus 23	1.92 × 10^5^	kPa
Young’s modulus 22	5.11 × 10^7^	kPa	Shear modulus 31	2.0 × 10^6^	kPa
Young’s modulus 33	5.11 × 10^7^	kPa	Volumetric response: shock Gruneisen coefficient	1.64	-
Poisson’s ratio 12	0.013	-	Parameter C1	3.57 × 10^3^	m/s
Poisson’s ratio 31	0.5	-	Parameter S1	1.3	-
Reference temperature	293	K	Specific heat	1.85 × 10^3^	J/kgK

**Table 6:** Material constants for Orthotropic yield strength.

Parameters	Value	Units	Parameters	Value	Units
Plasticity constant 11	0.016	-	Eff. plastic strain #1	0	-
Plasticity constant 22	6 × 10^−4^	-	Eff. plastic strain #2	0.01	-
Plasticity constant 33	6 × 10^−4^	-	Eff. plastic strain #3	0.1	-
Plasticity constant 12	0	-	Eff. plastic strain #4	0.15	-
Plasticity constant 13	0	-	Eff. plastic strain #5	0.175	-
Plasticity constant 23	0	-	Eff. plastic strain #6	0.19	-
Plasticity constant 44	1	-	Eff. plastic strain #7	0.2	-
Plasticity constant 55	1.7	-	Eff. plastic strain #8	0.205	-
Plasticity constant 66	1.7	-	Eff. plastic strain #9	0.21	-
/	/		Eff. plastic strain #10	0.215	-
Eff. stress #1	1.48 × 10^3^	kPa	Eff. stress #6	6.0 × 10^4^	kPa
Eff. stress #2	7.0 × 10^3^	kPa	Eff. stress #7	8.0 × 10^4^	kPa
Eff. stress #3	2.7 × 10^4^	kPa	Eff. stress #8	9.8 × 10^4^	kPa
Eff. stress #4	4.0 × 10^4^	kPa	Eff. stress #9	2.0 × 10^5^	kPa
Eff. stress #5	5.0 × 10^4^	kPa	Eff. stress #10	1.0 × 10^6^	kPa

**Table 7:** Numerical simulation results of projectile penetrating armor with two layers of PE.

*v* (m/s)	State of Perforation and Deformation	*p* (mm)	*v* (m/s)	State of Perforation and Deformation	*p* (mm)
700	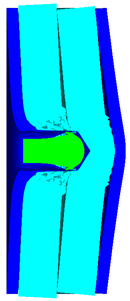	38.14	1100	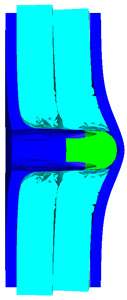	61.71
800	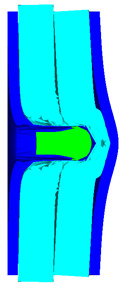	45.82	1200	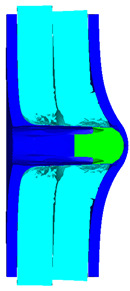	65.52
1000	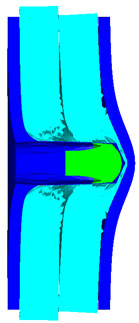	57.32	1300	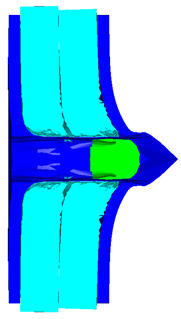	pass through

**Table 8:** Numerical simulation results of projectile penetrating armor with three layers of PE.

*v* (m/s)	State of Perforation and Deformation	*p* (mm)	*v* (m/s)	State of Perforation and Deformation	*p* (mm)
680	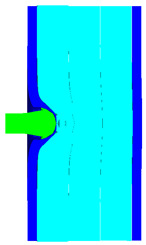	14.38	1190	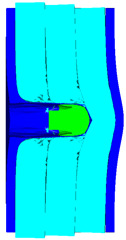	57.30
780	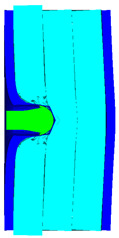	24.94	1300	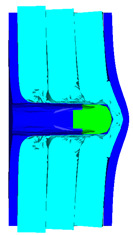	66.62
1000	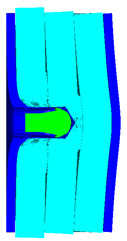	43.12	1400	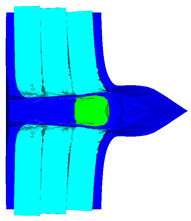	pass through

**Table 9:** Ballistic impact results of armor with two layers of PE.

Test No.	*v* (m/s)	Perforation State in the Front and Back	
1	759	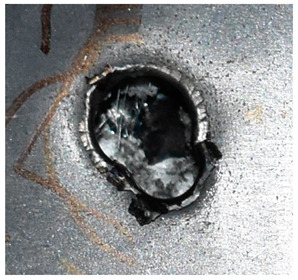	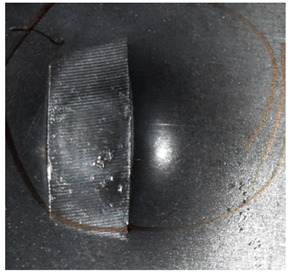
2	1139	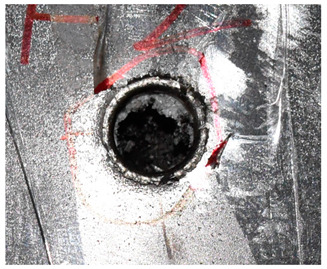	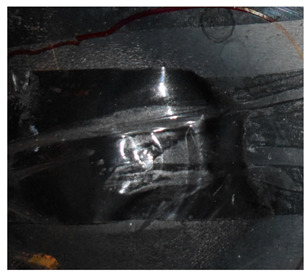
3	1174	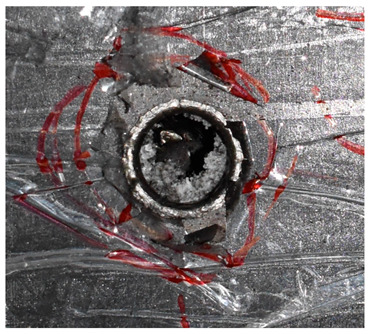	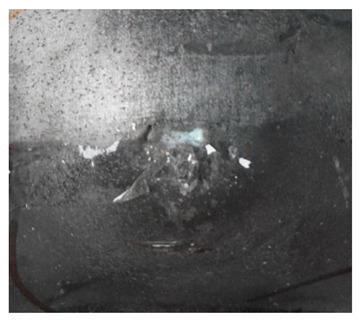
4	1174	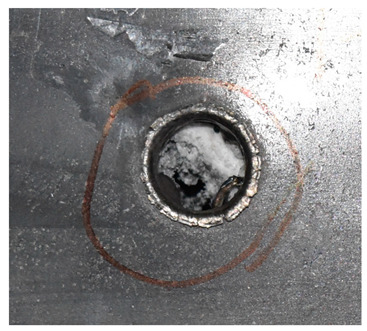	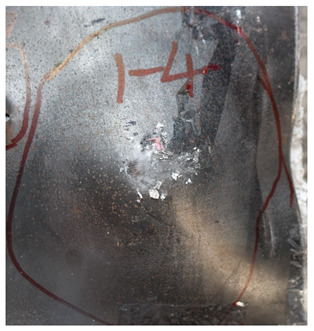
5	752	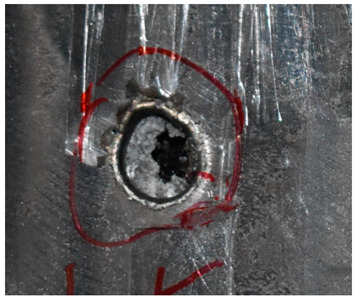	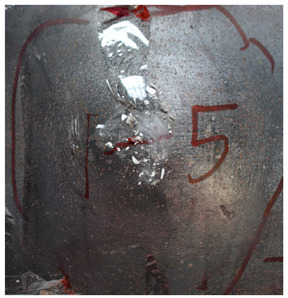

**Table 10:** Perforation dimension of armor with two layers of PE.

Test No.	1	2	3	4	5
Impact velocity *v* (m/s)	759	1139	1174	1174	752
Dimension (mm)	Φ21.02 × 45.02	Φ19.82 × 62.04	Φ19.96 × 64.07	Φ20.20 × 66.12	Φ19.20 × 44.28

**Table 11:** Ballistic impact results of armor with three layers of PE.

Test No.	*v* (m/s)	Perforation State in the Front and Back	
1	683	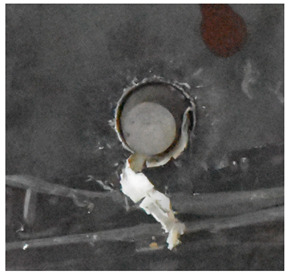	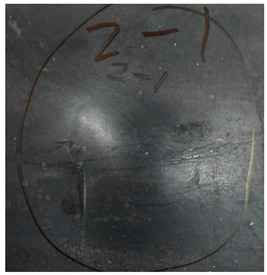
2	486	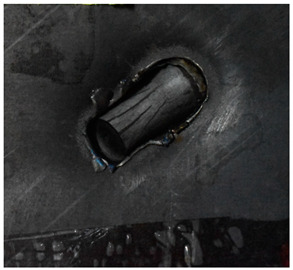	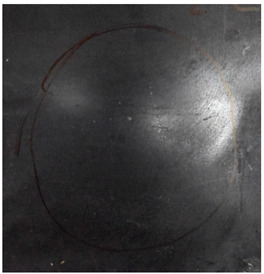
3	778	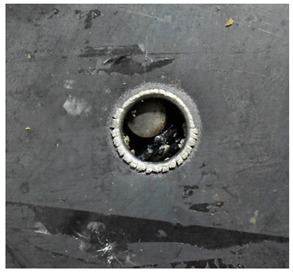	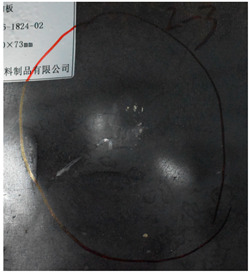
4	889	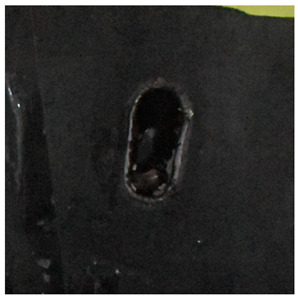	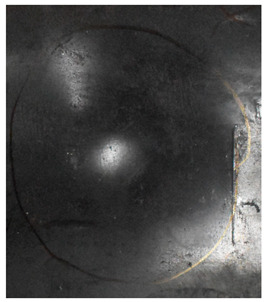
5	1175	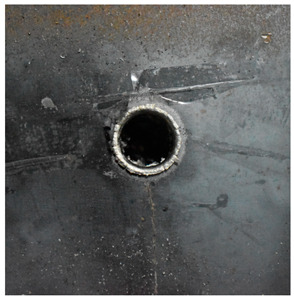	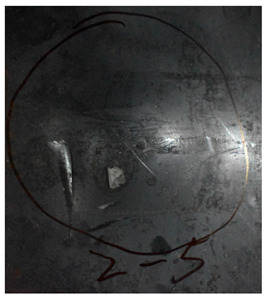
6	1190	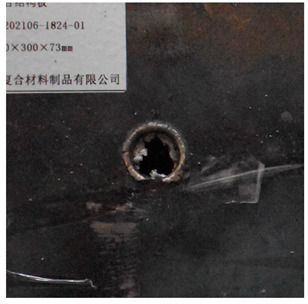	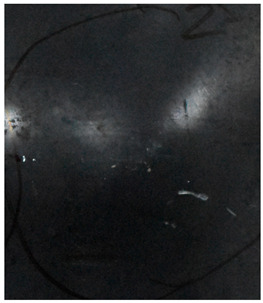
7	1227	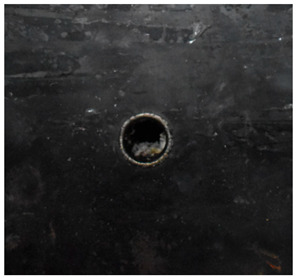	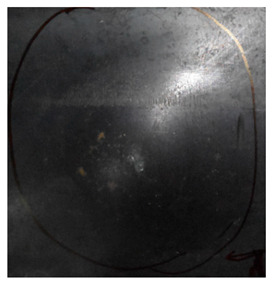
8	1304	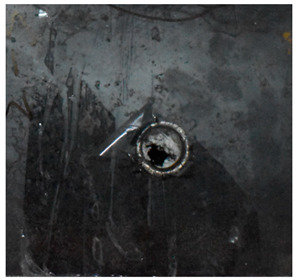	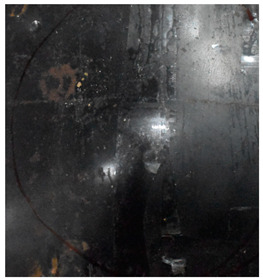

The nameplates on the armour shows parts of the information of the dimension and provider of the composite armours in Chinese.

**Table 12:** Perforation dimension of armor with three layers of PE.

Test No.	1	2	3	4
Impact velocity *v* (m/s)	683	486	778	889
Dimension (mm)	Φ24 × 12.39	/	Φ20 × 27.20	/
Test No.	5	6	7	8
Impact velocity *v* (m/s)	1175	1190	1227	1304
Dimension (mm)	Φ21 × 54.70	Φ21 × 57.01	Φ21 × 59.50	Φ21 × 65.20

**Table 13:** Depth of penetration for estimating through-thickness shear strength.

Armor Type	Impact Velocity *v*_i_ (m/s)	Depth of Penetration *p* (mm)	$\tau_{\max}\;(\mathrm{GPa})$
2 PE	752	45.02	7.30
	759	45.28	7.41
	1139	62.04	12.81
	1174	64.07	13.12
	1174	66.12	12.71
3 PE	683	12.39	20.42
	778	27.20	12.40
	1175	54.70	14.76
	1190	57.01	14.54
	1227	59.50	14.85
	1304	65.20	15.36

## Data Availability

The raw and processed data generated during this study will be made available upon reasonable request.

## Nomenclature

symbols	
*ρ*	density
*ρ*_0_	initial density
*P*	pressure
*v*_s_	shock wave velocity
*v*_p_	material particle velocity
*c*_0_	wave speed
*s*	a material-related coefficient
*C*	intercept of *v*_s_ − *v*_p_ curve
*μ*	a non-dimensional coefficient
γ_0_	coefficient of Grüneisen
*S*_1_, *S*_2_, *S*_3_	slope of the *v*_s_ − *v*_p_ curve
*a*	one-order correction of *γ*_0_
*σ*	effective yield stress
*ε*	effective plastic strain
$\dot{\varepsilon }$	strain rate rate
${\dot{\varepsilon }}_{0}$	quasi-static threshold strain rate
*A*	initial yield stress
*B*	hardening constant
*n*	work-hardening coefficient
*C*	strain rate constant
*m*	thermal softening exponent
*T*	temperature
*T_m_*	melting point
*Tr*	reference temperature
*T**	homologous temperature
*C_ij_*	coefficients of the stiffness matrix
$\varepsilon_{ij}^{d}$	deviatoric strains
$P\left(\varepsilon_{vol},e\right)$	volumetric strain
*v*	volume
*e*	internal energy
Γ(*v*)	Grüneisen coefficient
*P_r_*(*v*)	reference pressure
*e_r_*(*v*)	reference internal energy
*a_ij_*	plasticity coefficients
*σ_ij_*	stresses in the principal directions of the material
*k*	define the border of the yield surface
*S_ii_*	failure strength in the respective directions of the material
*D_ii_*	damage parameter
*L*	characteristic cell length
*ε_cr_*	crack strain
*G_ii,f_*	fracture energy in the direction of damage
*p*	depth of penetration
*v*	initial velocity
$E_{\mathrm{total}}$	total kinetic energy of the projectile
*m*_p_	mass of the blunt projectile
*v*_i_	impact velocity of the projectile
*E*_S_	energy absorbed in shear plugging
*E_B_*	energy absorbed in the bulging stage
$\tau_{Q235}$	shear strength of steel Q235
$\tau_{\max}$	effective through-thickness shear strength of the laminate
*r*_p_	radius of the hole
β	a non-dimensional multiplier
t_0_	thickness of the front sheet
t*_S_*	PE thickness penetrated through shear plugging
*m*_p_	mass of the projectile
*v_B_*	projectile velocity before bulging
*m_B_*	mass of the target involved in the bulging stage of PE
*m*_0_	mass of the target involved in the bulging stage back sheet
